# Dr. Hughes’ Address before the London (1881) Convention

**Published:** 1882-04

**Authors:** P. P. Wells

**Affiliations:** Brooklyn, N. Y.


### ADDRESS OF DR. HUGHES, PRESIDENT OF THE LONDON HOMŒOPATHIC CONVENTION.

P. P. WELLS, M. D., BROOKLYN, N. Y.

In the October number of THE HOMŒOPATHIC PHYSICIAN, we gave our views of some of the utterances of writers of papers read at this Convention, and of what seemed, from fragments of its reported doings, to be its probable outcome. For this we had but fragments, and on these we commented. How far a complete report would have modified our comments, if we had had one, we do not know, but, by the kindness of the President of that Convention, we now have before us his address, complete, and we return to it, not for the purpose of giving a complete review of this paper, but to comment on some portions of it, and to give some thoughts suggested by reading it, in the manner and spirit in which we would have discussed these had we been beside the author when this address was written.

Before taking up the first point with which we propose to deal, we would remark of the address, that in spirit and scholarly grace, it is just such as his friends and admirers in this country and elsewhere (and of these there are many), would have expected from his well known urbanity of character, and his uniform courtesy in his intercourse with friends and strangers. For these qualities the address is worthy of all praise. But excellent as these are, ever and in all places, there are interests of greater value, for which, if neglected or misrepresented, neither courtesy nor grace can be accepted as compensation or excuse.

The address, we may add further, is characterized by a liberality of spirit which will not fail to make it attractive to many readers. And just here we wish to say a word on this matter of "*liberality.*" It is a word often used, and often by those who use it with an air or expression of self-approbation, or even of self-congratulation, as if their conscious possession of this grace had raised them in their own estimation above all censure for whatever of shortcoming or transgression they may have committed. Now, *true* liberality of spirit is one of the loveliest of the characteristics of men, while that which is false is one of the ugliest. There is a true and a false in this matter, to which he who would build character wisely will do well to take heed. True liberality concedes to others the same right of judgment as to principles and practice which it claims for itself. The false would have it to be understood that differences in these are of no great consequence, that one is just about as good as the other, and that as between truth and falsehood it is not well to be too precise in our ideas and discriminations.

There has been more than enough of this. It has been put forth on the platform and in print, with an unblushing complacency which would seem to indicate that, to speaker and writer, truth and lies were just about equally worthy of our respect and confidence. Unlike this, true liberality contends earnestly and always for the *truth*, no matter who opposes, and insists that "no lie is of the truth." "*He is liberal*" has come to mean (and it would seem that it is expected this shall be accepted as certifying that he of whom it is said is a man to be approved of), that the man cares little for the difference between truth and falsehood. Is such a man to be approved of? If such a declaration be not too often a certificate of perfect worthlessness, we are greatly mistaken.

We have been moved to these remarks on *false* liberality, mainly, after a consciousness of their truth, by a memory of the frequent use made of a certain Chicago utterance of that noblest of noble-hearted men, our late honored friend, Carroll Dunham, as an apology for this, and nearly every other imaginable offense. That utterance has many times been made to do a work from which the spirit of its author would have shrunk with horror. With him there was no indifference as to the nature or results of truth and error. His whole life was an earnest, consistent advocacy of *truth*. He never appeared as the apologist of error. It was the good fortune of the writer to know Carroll Dunham intimately, from the time he entered upon his professional studies to his death. There were between us many, very many, confidential discussions, and among these, this matter of "*liberality*" was often the theme. I believe I know his views of the matter perfectly, as much so, probably, as any one now living, and I have no hesitation in saying he had no respect for that liberality which inculcates indifference in matters of faith and practice. At the same time, he was not accustomed to arraign his neighbor before the bar of his own judgment to answer in the matters of difference there might be between the two. He did not regard his neighbor as responsible *to him* for whatever of error he might have adopted or practiced. At the same time there was with him no indifference to or tolerance of error. He was ever an earnest advocate of the truth, as he was ever an example of one of its most successful practitioners.

In Dr. Hughes' address, p. 4, we read: "The thing we desire to propagate and develop, is the *method* of Hahnemann. It is a method—a mode of treating disease—this, and nothing more. It is not a doctrine or a system," etc. While we take issue with this wholly inadequate statement of what Homœopathy really is, we cannot refrain from the remark that he whose view of it is thus limited has no right to stand as its teacher either on the rostrum or in print. Homœopathy is much "*more*" than "*a method*." It is a philosophy of the relationship between diseases and their curatives. This makes up the *science of therapeutics*, and besides this we know no other. The

"*method*," which seems to be all the president sees of its beauty, extent and perfection, is only the *art* of the practical use of this philosophy. Here it seems to have been his misfortune that his view of his subject stopped, just when and where it should have gone on. And more than this, this philosophy is the unfolding and exemplification of a natural law. Hahnemann is sometimes spoken of as the founder of Homœopathy. This he was not. The founder of Homœopathy was the Creator of man. And the time when, was when the other natural laws were ordained. Hahnemann was only in a certain sense the discoverer of this natural law. It had been promulgated centuries before his day, had been opposed by those who seemed to think they knew all there was to be known, just like its opponents of to-day, and then it was forgotten. It was brought to Hahnemann's perception by the results of his own experiments and observation, and not by any record of an antique practice; so that, in a certain sense, he became its discoverer.

We maintain that this "method," this art of the president, is nothing less than one of the divinely enacted natural laws. We are not surprised, after reading this myopic vision of the law, limiting it to a mere "method," to hear him say, "We in no way determine how far its practical consequences shall reach."* Being, as it assuredly is, a natural law, these must "reach" just as far as the objects and relations with reference to which it was enacted extend, *i.e.* it must embrace *all* diseases and their curatives.

If this be a correct view, then *obedience* to this law becomes a *duty*, and cannot in any wise or in any degree become a mere matter of *choice*. The liberality which would thus dispose of it then is very like that which makes free with one's neighbor's goods, *i.e.* it becomes, as we have elsewhere said, a *crime*. The law is God's law, and not ours to dispose of as we may choose, to obey or repudiate at our pleasure. Not only does allegiance to its Author require this of us, but the best interests of the suffering sick as well. We believe no man as intelligent as to the records of practical Homœopathy as the author of this address undoubtedly is, will deny that the brightest and best of these are the outcome of strict obedience to the law and its logical corollaries. It is only needful to mention Hahnemann, Bœninghausen, Haynel and Hering, to make our meaning in this matter clear. In considering another portion of this address we may have occasion to refer again to these worthies and their record.

* Address, p. 6.

This being so, and the duty of the prescriber being to see to it that his patient receives from him nothing inferior to the *best*, whence comes the liberty claimed for him by the president, on page 6, to resort to "stimulants, sedatives, purgatives, caustics and counter-irritants," as do the Old School, regardless of law? If he knows no better, his duty is to learn the better way, to study that he may know more, rather than to turn to these resorts of a school which has in the use of them brooded in darkness these many centuries, and still refuses to come to the light. The liberty claimed on this page is a liberty to prefer darkness to light, and this certainly is not a privilege to be very highly esteemed. It dooms the practitioner to a perpetual ignorance of the better way, and his patients to a loss of the benefits the better way (the way of law) has in store for their relief. The only semblance of good to any man or interest that can come from this liberty is, it seems, for the time, to save the practitioner the labor involved in a compliance with the requirements of law. This liberty of choice, while it saved him from a labor which he lacked the force of will to undertake, dooms him to the imbecility which is the inevitable result of a refusal to use the powers and opportunities God gives to us.

We can hardly think well of the claim here set up for a liberty to do thus badly, when results so disastrous to all concerned are so certain to follow its acceptance and practical use. We have known practitioners, who called themselves homœopathists, who were accustomed to these resorts "when they thought fit," but, whatever they may have been as members of society, as men—as homœopathists they were only as the least of all flesh. The president seems to have had more than an inkling of this result even while making this claim, and this is even more clearly expressed on page 7.

On page 10, the president says: "The fact is that there are (as I endeavored to show in 1877) two homœopathies, * * * but they belong to different stages of his (Hahnemann's) career. The first dates from 1796—1812. It is that of the Hahnemann of the *Medicine of Experience* and the *Fragmenta de Viribus*, of the first edition of the *Organon*, and the first edition of the *Materia Medica Pura*. * * *. When I first spoke of two homœopathies it was mainly to urge upon my colleagues that the later thoughts of Hahnemann—the Hahnemann at any rate of 1812 to 1828—were worthy of attention," etc. We are not surprised that so intelligent a man as our president should so have regarded these thoughts, nor to hear him say he "would do so again on fit occasion." Could any "occasion" have been more "fit" for this than that of this convention over which he was the head?

But how "*two* homœopathies"? We do not regard the expression as felicitous, though we think we get the president's meaning from it. There were matters in Hahnemann's later teachings not found in his earlier. But this certainly can hardly be equal to transforming the teachings of the master, at the different periods of his life, into two entities, differing from each other essentially. The difference was of *growth*, not of essential distinction of entity. To say, because of this growth, there were two distinct individualities, would be like saying Richard Hughes at 5 and Richard Hughes at 50 years of age were two distinct individuals. Growth does not destroy identity, As the man with the increase of stature gains new knowledges and develops new faculties, so the science of therapeutics as developed by the master, added new elements after its birth, till it had reached the more perfect stature of its present existence, the added principles and corollaries not changing its identity, but only bringing, so to say, new faculties for its practical life and successes.

The president gives some of these products of the master's more mature thoughts and labors, which he says are "conspicuous by their absence" in the earlier announcement of the homœopathic doctrine, viz.: "the vital force" (which, by the way, we did not know till now told in the address, p. 10, is only a product of Hahnemann's speculation), "the psoric origin of chronic diseases, the dynamization of medicines"—these are some of the later thoughts which the president would again commend to his colleagues as worthy of their attention, on a fit occasion. It is well he should do so, as it is a lamentable fact that very many of these, in their acceptance of Homœopathy, seem never to get beyond that first stage in which its development was obviously so incomplete. Their wisdom in stopping thus far back and rejecting subsequent developments, is as though our worthy and esteemed friend at fifty should utterly refuse to employ the faculties and powers he has acquired, with so much honor to himself, since his age of five. And more than this, it will be well for these colleagues if he succeeds in thus calling their attention to these later discovered elements of the homœopathic philosophy, for it has been by the knowledge of these, and by a practice in which these were made controlling factors, that the brightest records of practical healing have been achieved. Small wits have sneered at the three chronic miasms; Bœninghausen believed in them, and by the light of this faith he gained a success in the treatment of chronic diseases, which gave him a position as a successful prescriber, which it is believed no other man, of whatever faith or practice, has ever attained. His faith in these miasms led to the study of them with earnest carefulness, and this, to a knowledge of them which contributed so largely to make him the master in healing he was. Meaner men may ridicule this faith; indeed, very mean men have done this often; but they, nor those who have been content to stop at the *non credo* of the skeptic, have ever had the courage to compare records of success with this peerless prescriber. All those elements of the homœopathic philosophy which are habitually spoken of as Hahnemann's "theories," his "errors," or even as his "fanaticisms"—this master had proved them all, as he thought, to be irrefragable truths, and these truths it was which gave him his power as a prescriber, which certainly was something quite unique. The same is true of the other heroes, his cotemporaries in practice, who contributed so largely to give this philosophy and practice its present world-wide reputation and acceptance.

When we say it was the acceptance and practical use of these often ridiculed, and oftener rejected, elements of the homœopathic philosophy, which constituted the one great characteristic of Bœnninghausen's practice, we speak from personal knowledge. His faithful adherence to them, to the utmost detail, even after nearly half a century of practical prescribing, was something little less than wonderful. No repetition of a duty, however often, begat with him the least carelessness, nor brought any temptation to seek, from this familiarity, any shorter or easier way than that through the "totality of the symptoms," and their most careful comparison with the record of the materia medica. At 76 he was equally careful and laborious as at the beginning; and this care and labor we have seen followed by results the most surprising and gratifying.

Is there not in such an example of faith and faithfulness a lesson for those who are now professed believers in the philosophy which dominated his practical career? Is there not a duty for them to emulate his successes? They may assuredly know that these can only be realized by implicitly following his example of faith and practice. This can come from no partial acceptance of homœopathic philosophy, which so many now seem tempted and encouraged to adopt. Partial acceptance or partial faithfulness can only result in partial success at the best, and will, but too often, and too likely, end in complete failure, and resort to old school abominations. For it should be remembered, that in our school these are never met, except in the practice of partialists, and that Bœnninghausen and those of like practice have never felt the slightest need of them.

The president gives warning, that "when the volume we shall issue comes to be read, it will be found to be wholly and powerfully representative" of this partially developed Homœopathy of the early period. Perhaps it was this partial character of the Homœopathy represented in the convention which made the fragments of the reported doings so unsatisfactory to those who looked for something better as its outcome. Was it the imbecility of infancy?

On page 7, our president gives utterance to a most important idea: "We ought by this time to be able to define *what are the exceptions* [italics ours] we recognize to the rule 'let likes be treated by likes;'* for, like every other rule, it has its exceptions," etc. We repeat, with hearty concurrence, that it is full time that those who know of such exceptions, inside of the circle of curable diseases, should make known to the world what they are. For ourself we freely and earnestly say, that within this circle we know of no such exceptions. As to incurable diseases, and alas there are such and but too many of them, it is of little use to talk of "rules" or "exceptions." And, further, we most heartily join our president in his declaration that "outside these limits [*i.e.* of these exceptions] to obey it [the rule] implicitly." This is sound doctrine, and as we understand the matter, calls for implicit obedience to the "rule" in all cases of curable sickness till these "exceptions" are clearly stated and fairly proved to be "exceptions."

And further, on page 5, the address says, "we do not listen to those who would have us cut ourselves adrift from him [Hahnemann] so as to profess a Homœopathy freed from the errors and fancies with which he has weighted it." It would seem to have been easy, for a long time, for men to talk of the "errors and fancies" of the great master. This has been and is quite a cheap kind of talk. It would have been better if these men had, while so talking, plainly

* That the president has not been "able" to do this, nor any one of the many who have practiced as though such "exceptions" really existed, not one of them, after so long a time, does it not go far as proof that no such exceptions have place except in the imagination of these partialists? These should be either brought to light and demonstrated, or the talk of them as recognized realities should stop.

stated these "errors and fancies," that the rest of the world might know just what the points are to which they applied these words, that there might be a fair opportunity for judgment as to whether these so-called "errors" may not be important *truths*, rather. This is certainly possible, and there are not a few who heartily believe that with this opportunity many, if not all, will be found of this better character. We are sure that till this judgment is so had, our friend does well to decline this "*cutting adrift.*"

And further, still, on page 9, there is given us another opportunity to agree with our friend, which is always pleasant. It is in these words: "I am not prepared to throw overboard, merely to lighten the ship, the wealth of experience gained and recorded in the past as the result of infinitessimal medication." To do this would be only paralleled by the folly of "throwing overboard" the steamer's engine for a like purpose. It would be dispensing with a chief good for the purpose of "lightening a ship" which really needs no relief of the kind. It is well, therefore, that he so decidedly declines to part with this "wealth," and the more so, as it is just this experience which has given to Homœopathy its fame, and in which is found its chief triumphs. It is neither the office nor the result of a partial Homœopathy, this of successful aggression against the practice and prejudices of three thousand years. The feebleness of undeveloped life is not the instrument with which to engage in so important a work. And it is just this feebleness which partialists accept when they refuse to go beyond its early announcements in their faith and practice. It is not wonderful that feeble ones should sometimes fear for the safety of the ship so freighted. Not so our president, and he is right. That which the feeble ones fear is just the power which will carry the good craft onward to a triumphant termination of its voyage. It is not to be "thrown overboard."

There are in this address many noble utterances of which it is more than pleasant to approve, and much of expression of generous spirit with which all good men will sympathize. Many of these have been marked for comment, but the length to which this paper has come, reminds us that it is nearing the limits which can be given to it in the quarter to which it is destined. We shall refer to but one more, and then be done.

On page 14, the president says: "I love and sigh for unity—[*i.e.*, for homœopathists with allopathists], but there is something I prize even dearer still, and that is truth." There are many, there can be no doubt, who will sympathize with this desire. We are not of the number, we can see no gain in a unity of error with truth. There can be no gain to error—there can be only damage to truth, in such a union, if indeed this were possible, which it is believed it is not. Our president believes it possible, and of certain attainment in some far-off future. We do not share in this faith. Some years since we heard a valued friend express the hope that he should live to see the two schools "united on a scientific basis." This, of course, meant a unity as to the science of therapeutics. The hope was certainly a very sanguine one, or he was looking for an uncommon longevity. If he expected old school men to come over in a body on to our ground, (we have no objection to this), he was a very sanguine man, and it might not have been impertinent to inquire on what his hope was based. Certainly union in this case would imply compromise, a giving up on the one side and the other of something each held to be more or less important. Now, as we hold Homœopathy to be the science of therapeutics, and as we know of no other, that it is a truth in whole and in part, what is there of this the believer in it can give up? If the prophecy should be realized in our day, it is to be feared it would turn out but badly for the writer of this and our president, for assuredly, in these and all other circumstances, we mean to cleave to the truth in its integrity.

Those who are hopeful as to the near approach of this professional millennium may have taken some courage to their hope from the recent action of the New York State Medical Society.

That honorable body, in fashion quite magnanimous, at their recent session in Albany, removed the ban which forbade its members to consult with homœopathists. Why they should have done this just now, after all these years of attempted ostracism, is not very apparent. Did anybody request this at their hands? Homœopathy is now the very thing it has been through all these years when it has been the thing they have hated most, and its practitioners are neither better nor worse than they have been through all these years, when they have omitted no opportunity to treat them with the utmost contempt, and when a consultation with them was expulsion from fellowship. Why then this recent action of this respectable body? We confess we can see but one reason for this removal of this very harmless ban. We suggest it only as a possible reason. We can imagine that the long-time refused consultation, which nobody desired or asked for, was at last discovered to have put the members of this society into rather an awkward position before the public. To escape from this may have been a motive. But then what of the liberality which some think they seem to see in this wholly uncalled for act, which nobody needed or desired, at least outside their own ranks?

While some think they see a liberality in the spirit of this body disclosed by this act, others think they see only a new form of attempt to give Homœopathy its death blow. They think the members of this society reasoned somewhat in this way: We have tried to kill it by active opposition and abuse these many years, and yet it has grown and flourished. This method has proved a failure. Now let us try the other tack. Let us show favor and patronize it, and it will die the speediest. Did they mistake Homœopathy for a new form of patient? It certainly proposes a new form of allopathic practice.

We have said nobody asked for professional consultations with the members of this society. We add, we believe no one has felt the least need of them. Their advice as to the administration of our law of therapeutics could only be perfectly useless, if not positively harmful, and this for the reason that they know absolutely nothing at all about it. Our advice to them could only be to use the like which cures, and point it out to them, and see how quickly they would accept it. The writer has been something more than forty years endeavoring his best to obey the law of cure he has accepted and trusts, and in all this time he has no recollection of once feeling that any adherent of the school in which he was educated could help him the least in any difficulty he has encountered. We repeat then, why this repeal? We might be impelled by prudence into a memory of the Trojan utterance—*Timeo Danoas, et dona ferentes,* if we did not at once remember these old school gentlemen *bring us no gifts.* They never loved Homœopathy or its advocates less than now. If this repeal has any influence to lessen professional rancor, we can all rejoice in the result.

---

THE next Annual Convention of the Western Academy of Homœopathy will be held at Kansas City, June 20—22. Arrangements are being completed for a grand excursion to Denver and return at greatly reduced rates; other attractions will be offered to make the occasion a memorable one.

C. H. GOODMAN, M. D.,
*Genl. Secry.*, 2619 Pine Street, St. Louis, Mo.

